# Long-term gas exchange characteristics as markers of deterioration in patients with cystic fibrosis

**DOI:** 10.1186/1465-9921-10-106

**Published:** 2009-11-12

**Authors:** Richard Kraemer, Philipp Latzin, Isabelle Pramana, Pietro Ballinari, Sabina Gallati, Urs Frey

**Affiliations:** 1 Department of Paediatrics, University of Berne, Inselspital CH-3010 Berne, Switzerland; 2 Division of Paediatric Respiratory Medicine, Department of Paediatrics, University of Berne, Inselspital, CH-3010 Berne, Switzerland; 3 Division of Human Genetics, Department of Clinical Research, University of Berne, CH-3010 Berne. Switzerland; 4 Institute of Psychology, University of Berne, Muesmattstr. 45, CH-3000 Bern Switzerland

## Abstract

**Background and Aim:**

In patients with cystic fibrosis (CF) the architecture of the developing lungs and the ventilation of lung units are progressively affected, influencing intrapulmonary gas mixing and gas exchange. We examined the long-term course of blood gas measurements in relation to characteristics of lung function and the influence of different *CFTR *genotype upon this process.

**Methods:**

Serial annual measurements of PaO_2 _and PaCO_2 _assessed in relation to lung function, providing functional residual capacity (FRC_pleth_), lung clearance index (LCI), trapped gas (V_TG_), airway resistance (sR_eff_), and forced expiratory indices (FEV_1_, FEF_50_), were collected in 178 children (88 males; 90 females) with CF, over an age range of 5 to 18 years. Linear mixed model analysis and binary logistic regression analysis were used to define predominant lung function parameters influencing oxygenation and carbon dioxide elimination.

**Results:**

PaO_2 _decreased linearly from age 5 to 18 years, and was mainly associated with FRC_pleth_, (*p *< 0.0001), FEV_1 _(*p *< 0.001), FEF_50 _(*p *< 0.002), and LCI (*p *< 0.002), indicating that oxygenation was associated with the degree of pulmonary hyperinflation, ventilation inhomogeneities and impeded airway function. PaCO_2 _showed a transitory phase of low PaCO_2 _values, mainly during the age range of 5 to 12 years. Both PaO_2 _and PaCO_2 _presented with different progression slopes within specific *CFTR *genotypes.

**Conclusion:**

In the long-term evaluation of gas exchange characteristics, an association with different lung function patterns was found and was closely related to specific genotypes. Early examination of blood gases may reveal hypocarbia, presumably reflecting compensatory mechanisms to improve oxygenation.

## Background

Mixing of inspired gas is a prerequisite for effective respiration and is dependent upon the architecture of the lung. In children with cystic fibrosis (CF), the architecture of the developing lungs and the ventilation of peripheral lung units are progressively affected, influencing the efficiency of gas mixing and gas exchange. We have previously reported observations that inequalities in ventilation occur significantly earlier in the course of lung function decline than changes in other functional characteristics [1]. Moreover, we have recently demonstrated in this *Journal*, that pulmonary hyperinflation and development of trapped gas represent major functional features of disease progression in children with CF [2]. In agreement with several author groups, airway dysfunction and pulmonary deterioration in CF is thought to be expressed by ventilation inhomogeneities [1-6], pulmonary hyperinflation [2,7] and gas trapping [2,8,9] occurring very early in life [5,10,11]. The different patterns of functional derangements stress the need to include a range of lung function tests including gas exchange characteristics and to investigate whether the *CFTR *genotype has an impact upon these processes in CF patients, as has been previously reported [12-14].

Limited knowledge exists as to whether gas exchange parameters measured under resting conditions are helpful in defining progression of lung disease in children with CF. A correlation between survival and adequacy of gas exchange, and the hypothesis that carbon dioxide retention may be a predictor of survival, was initially published by Wagener et al. in 1980 [15]. Mechanisms of impaired gas exchange and their influence on ventilation-perfusion inequality through shunts have recently been studied by Soni et al. [16]. A previous study of ours performed in a limited number of children with advanced stages of CF showed the interdependence of oxygenation, ventilation inhomogeneities and trapped gas assemblage [8].

The aim of this prospectively acquired cohort study was to evaluate the onset and course of deterioration of gas exchange in relation to changes in lung volume, ventilation distribution, trapped gas and airway obstruction, as well as the influence of specific genotypes in CF patients, from the 5th to the 18th year of life. Following up on our recent work published in this *Journal*, we looked at the long-term course of gas exchange characteristics in relation to different facettes of lung function [1,2].

## Study population and methods

### Bernese Cystic Fibrosis Patient Data Registry

Patients were recruited from the Bernese Cystic Fibrosis Patient Data Registry, prospectively developed as an extension of the American Cystic Fibrosis Patient Registry founded by Warwick in 1966 [17]. Standard clinical and biomedical parameters, as well as gas exchange characteristics and lung function data of a total of 204 CF patients regularly seen at the outpatient clinic between 1978 and 2008 are documented in the registry. The inclusion criteria for the present study were: (*i*) CF diagnosis based on the presence of characteristic phenotypic features [18], (*ii*) confirmed by a duplicate quantitative pilocarpine iontophoresis sweat test measuring both Na and Cl values > 60 mEq/L as well as by (*iii*) genotype identification using extended mutation screening of both alleles [19,20], and (*iv*) complete documentation of a minimum of 3 blood gas analyses with concomitantly measured lung function parameters performed annually between the ages of 5 to18 yrs, investigated during clinical stability. Twenty of the 204 patients (9.8%) were younger than 6 years of age (blood gas analysis and lung function data not available), and in 6 CF patients (2.9%), fewer than 3 annual measurements were available. Some of lung function data in the database have been reported previously with respect to the phenomenon of ventilation inhomogeneities [1] and pulmonary hyperinflation [2], and the sensitization against *Aspergillus fumigatus *[21]. There is no overlap with previous publications with respect to the major topic of the present report dealing with gas exchange characteristics. This study protocol was approved by the Departmental Ethics Committee of the University Children's Hospital Berne and by the Government Ethics Committee of the State of Berne, Switzerland.

### Pulmonary Function Measurements

Whole-body plethysmography and the multibreath nitrogen washout (MBNW) technique provided data pertaining to functional residual capacity (FRC_pleth_, FRC_MBNW_), lung clearance index (LCI), volume of trapped gas (V_TG_), effective specific airway resistance (sR_eff_), and forced expiratory indices (FEV_1_, FEF_50_). Measurement techniques have been described in detail in previous papers [1,2,21]. All values were expressed by z-transformation in standard deviation scores (SDS), based on gender- and age-specific regression equation [22-25], specifically calculated for each lung function device as previously presented [1,2]. Full details concerning lung function techniques, calculation of SDS, and the statistical methods are given in the additional file 1.

### Blood Gas Analysis

In children, the preferred technique for routine blood gas analysis is the sampling of arterialized capillary blood from the earlobe [8,26,27], a technique that has been established for clinical use and applied in various long-term studies [28-31]. The accuracy of this technique depends upon careful preparation of the earlobe [26], puncture technique [26,27] and immediate analysis. Several author groups have validated the accuracy of this particular technique for clinical and long-term evaluation of gas exchange [26,27,32-34], provided certain important methodological conditions are fulfilled. Therefore, oxygen (PaO_2_) and carbon dioxide (PaCO_2_) tensions were measured in arterialized blood collected from the ear lobe [8,26,27,33] using a Radiometer ABL5, Copenhagen, Denmark.

Blood gas sampling was performed after whole-body plethysmography and before the multibreath nitrogen washout measurements. The procedure was done in a quiet atmosphere and our patients were familiar with the process. The ear lobe was prepared according to the technique initially described [26,27], and previously established in our laboratory [8]. Vasodilatation of the earlobe was achieved by rubbing the lobe with a nicotinate paste (Finalgon) [26,27,32,33] for at least 10 minutes and heating with an infrared lamp. During quiet breathing, the arterialized blood was collected from a drop on the inferior aspect of the earlobe, from which it was drawn into 3 thin heparinized glass capillary tubes by surface tension under the guidance of a gloved finger over the open end of the tube. The capillary tubes were then kept on ice until aspiration into the gas analyzer, which was carried out immediately after the blood draw.

### Definitions of Gas Exchange Disturbance at Rest in Children

Details of the blood gas analysis performed by earlobe puncture technique [8,26,27], and information on reproducibility are given in the additional file 1. Most *definitions of hypoxemia *are related to arterial blood gas values obtained during exercise testing predominantly performed in adult patients. There is no clearly defined cut-off indicating gas exchange impairment at rest in children with lung disease. Lamarre et al. [35], as well as Stokes et al. [36] defined hypoxemia at rest in children as a PaO_2 _<80 mmHg. Gaultier et al. suggested defining hypoxemia in children as less than 90% of predicted values [27,37]. We re-calculated the original data of Gaultier et al. using a power function based on age in years and taking the 90 % level of confidence of Gautier's predicted values. Accordingly, the lower limit would be 78.3 mmHg for children aged 6 years, and 81.9 mmHg for children aged 8 years [27,37]. We therefore took a PaO_2 _level of 80 mmHg as the lowest limit defining sufficient oxygen uptake [27].

### Anthropometric data

Heights and weights were measured by experienced nurses, using a Harpenden stadiometer and according to recommended procedures [38]. The computation of longitudinal weight and height data was based on calculation of age- and gender-corrected body mass index (BMI) expressed as *z*-scores, using the method and values for children reported by Cole et al [39].

### Genotype Analysis

All patients were genotyped by a SSCP/HD scanning method followed by direct sequencing of the variants, thus permitting rapid and sensitive detection of 97-98 % of known and novel (newly identified) CF mutations, as previously described [19,40]. Genomic DNA was extracted from EDTA blood samples using the QIAamp Maxi Kit (Qiagen) according to the manufacturer's recommendations and quantified by spectrophotometry. In addition, a non-invasive method of buccal cell brushing [20] was used to obtain DNA from premature infants, recipients of previous blood transfusions and infants with me meconium ileus. Mutation screening of the entire coding sequences of the *CFTR *gene (including the 27 exons and exon/intron boundaries, intron 11 and 19, as well as the promoter region) was performed in each patient using a well-established single strand conformation polymorphism/heteroduplex (SSCP/HD) analysis.

### Data Computation and Statistical Evaluation

Repeated serial measurements of lung function data, acquired annually, were calculated as annual mean ± SEM changes for presentation, and the statistical analysis included procedures such as univariate regression procedures, linear mixed model (LMM) analysis [41], and binary logistic regression analysis, using SPSS (version 17, SPSS Inc., Chicago, USA). Graphical presentation was completed using Prism software (version 4.0, GraphPad Software, Inc., San Diego, USA). In order to avoid potential confounding factors such as age at diagnosis, improvement of management and/or treatment over the years of observation as described by Soni et al [16], we assessed the influence of these potential confounders by 3 characteristics such as "year at birth", "year at diagnosis" and the "year at testing". The "year at test" proved to be the principal confounder influencing the course of PaO_2 _significantly (*t *= 2.831; *p *= 0.005). Consequently, "year at test" was taken as a covariate in all statistical analyses.

### Linear Mixed Model Analysis (LMM)

The application of linear mixed model analysis evaluating repeated measurements has several advantages. In contrast to General Linear Models (GLM), the time points at which measurements are obtained need not be equal for all subjects, and therefore, subjects with missing data are not dropped from the analysis. In addition LMM analysis features a single model for a repeated measurement approach (*i*) assessing influences of various effects, (*ii*) estimating these effects at each time point, and (*iii*) comparing them correctly against a background of between-patient variation. There is no need to calculate mean values across all time points (to obtain the overall effect influences), or to analyze time points separately (to obtain specific effects at each time point). Standard errors for effects at individual time points are calculated using information from all time points and are, therefore, more robust than standard errors calculated from separate time points. Finally, the covariance pattern of the repeated measurements can be determined and taken into account (e.g. the model can figure out whether measurements are correlated across all time points, or whether they show more complex pattern over time) [42]. These are important advantages especially for observational studies, where people often miss regularly scheduled clinic appointments or experimental sessions. In addition, LMM analysis offers the possibility to select the best variance-covariance structure for repeated measurement data, and interactions of interest can be specified [43]. More details to the discussion LMM versus GLM analysis are given in the additional file 1, under the heading "*Options of Statistical Modeling*".

## Results

### Characteristics of Study Population

The inclusion criteria were fulfilled for a patient cohort of 178 CF patients (88 males; 90 females) recruited from the Bernese CF Registry (87.3%). Their follow-up statistics and the distribution of CFTR mutations are given in Table 1. From the entire database, 26 patients (12.7%) were excluded from evaluation because of insufficient number of tests (<3 per year) or an age younger than 5 years. Of these 178 CF patients, 14 (6.9%) died during the observation period with a mean age (range) at death of 16.4 ± 5.9 (8.6 to 24.2) years. One girl died in a car accident and the remaining patients had a PaO_2 _of 56.5 (49.4 to 63.1) mmHg, and a PaCO_2 _of 40.8 (36.2 to 46.8) mmHg in the last year of life. The individual follow-up data of these CF patients who died during the observation period are given in the additional file 1.

**Table 1:** Patient cohort (A), data base characteristics (B), and distribution of *CFTR *mutations (C) taken from the Bernese CF Registry (n = 178, 87.3% of a total number of 204 CF patients)

A						
	Patient cohort follow-up statistics					
	**Gender distribution of patients**			**Blood gas tests within age periods**		

		**n**	**%**			

	- males	88	49.4	5 to 8 y	427/1457	29,3%
	- females	90	50.6	9 to 14 y	527/1457	36.2%
		178	100	15 to 18 y	503/1457	34.5%
	From entire database, 26 patients (12.7%) excluded because of insufficient number of tests, (6) or age < 6 years (20)					
						
**B**						
	**Blood gas test and lung function measurement follow-up statistics**					
	**Number of blood gas tests median (range)**			**Blood gas tests per year of observation**		
	Total of tests	1457		1987 to 1993	326/1457	22.4%
	per child	8.1 (3-15)		1994 to 2000	539/1457	37.0%
	per year of observation	68.2 (37-90)		2001 to 2008	592/1457	40.6%
						
**C**						
	**Distribution of CFTR mutations**					
				**n**		**%**
						
	Inframe/inframe (F508del[2])		a	103		57.9
	Inframe/nonsense		b	22		12.4
	Frameshift/F508del		c	19		10.7
	Frameshift/non-F508del		d	12		6.7
	Inframe/splicesite		e	7		3.9
	Miscellaneous		f	15		8.4
	Total			178		100.0
	Equal distribution of CFTR genotypes over age range and over years of observation					

*CFTR*: cystic fibrosis transmembrane regulator

All patients except 3 suffered from pancreatic insufficiency. *P. aeruginosa *infection was present in 111 patients (67.7 %), with a mean age at onset of 5.7 years (range 0.1 to 20.5 years). Twenty-three patients were intermittently colonized and 30 remained free from *P. aeruginosa *colonization. Five patients underwent lung transplantation and in 2 the liver was transplanted. Within this study cohort of patients, a total of 1457 tests were performed representing a median (range) of 8.1 (3-15) test sets per child, or 68.2 (37-90) test sets per year of observation. Gender was equally distributed. According to the frequencies in our population-specific *CFTR *genotype distribution, the patients were stratified into 6 groups consisting of (*a*) F508del homozygotes F508del[2| (inframe/inframe): n = 103 (57.9%), (*b*) R553X, G542X, Q525X and E585X compound heterozygotes with F508del (inframe/nonsense mutations): n = 22, (12.4%), (*c*) 3905insT compound heterozygotes 3905insT/F508del (frameshift/F508del): n = 19, (10.7%), (*d*) 3905insT compound heterozygotes with other than F508del (frameshift/non-F508del): n = 12, (6.7%), (*e*) 1717-1G>A, 621+1G<T and 4005+1G>A compound heterozygotes with F508del (inframe/splicesite): n = 7 (3.9%), and (*f*) miscellaneous genotypes n = 15, (8.4%). The genotypes were equally distributed over the age-range studied and the years of observation.

### Deterioration of Gas Exchange Characteristics related to Age

**Figure 1:**
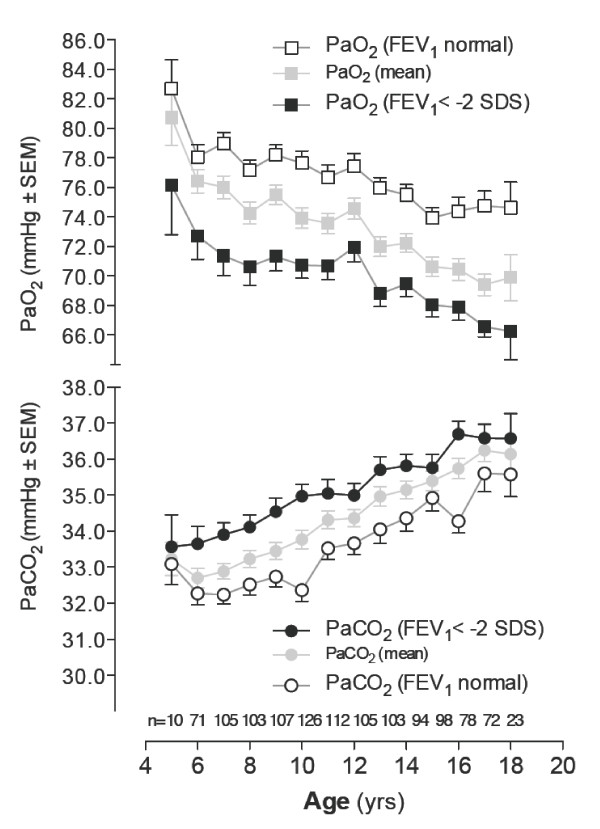
**Annual changes of gas exchange characteristics (PaO_2_, PaCO_2_) shown as mean ± SEM of repeated annual measurements over the age range 5 to 18 years (halftone symbols), stratified into measurements concomitantly obtained under normal FEV_1 _> -2 SDS (open symbols) and abnormal, FEV_1 _< -2 SDS solid symbols**. Measurement numbers (n) are given along the x-axis.

In Figure 1 age-related annual changes of PaO_2 _and PaCO_2 _(mean ± SEM) are shown, and in the following section some aspects of hypoxemia, hypercapnia, as well as a new phenomenon of hypocarbia are presented.

*Hypoxemia*. Overall PaO_2 _presented with a linear decline from a mean ± SEM of 80.7 ± 1.9 mmHg at 5 years of age to a mean of 69.9 ± 1.6 mmHg at age 18 years. As FEV_1 _is the most commonly used lung function parameter internationally, the figure additionally shows the PaO_2 _decline within 2 subgroups stratified according to normal FEV_1 _(z-score > -2 SDS) or abnormal FEV_1 _(z-score < -2 SDS). The measurements within the 2 subgroups were equally distributed over the age range studied. Unpaired *t*-test with Welch' correction compared PaO_2 _measurements with normal FEV_1 _(46.8%) presenting a mean ± SEM oxygenation of 76.9 ± 0.62 mmHg versus PaO_2 _measurements with a FEV_1 _<-2 SDS with a mean oxygenation of 70.2 ± 0.70 mmHg This significant difference in PaO_2 _decline was mainly due to a mean difference of 6.7 ± 0.94 mmHg between the FEV_1_-subgroups (*p *< 0.0001) and not to a difference in their slopes.

The annual changes in PaCO_2 _demonstrated a more complex course. The mean PaCO_2 _of 33.2 ± 0.45 mmHg at 5 years of age showed a transient stagnation in a large proportion of patients before rising to a mean of 36.1 ± 0.47 mmHg at the age of 18 years. This phenomenon of low PaCO_2 _measurements was observed in 103 of the 164 patients (62.8%), mainly associated with a FEV_1 _> -2SDS, and occurred over an age range of 5.8 to 15.8 years. An unpaired *t*-test with Welch' correction comparing PaCO_2 _measurements with a mean PaCO_2 _of 33.7 ± 0.32 mmHg for blood gas measurements associated with normal FEV_1 _versus a mean PaCO_2 _of 35.1 ± 0.29 mmHg in association with abnormal FEV_1 _also revealed a significantly different PaCO_2 _course with a mean difference of 2.4 ± 0.6 mmHg between the FEV_1_-subgroups (*p *= 0.0002).

*Hypercapnia*, defined as a PaCO_2 _>45 mmHg at rest, was observed only in a few patients in our collective. In contrast, we were surprised by some very low PaCO_2 _values occurring during the course between ages 5 to 12 years (Figure 1).

*Hypocarbia*, and especially a lower level for PaCO_2 _at rest in children has not yet been defined internationally. Using three different statistical techniques (binary logistic regression, ROC and discriminant analysis) explained in detail in the additional file 1, hypocarbia was defined as a PaCO_2 _less than or equal to 34 mmHg.

### Associations with Genotype

**Figure 2:**
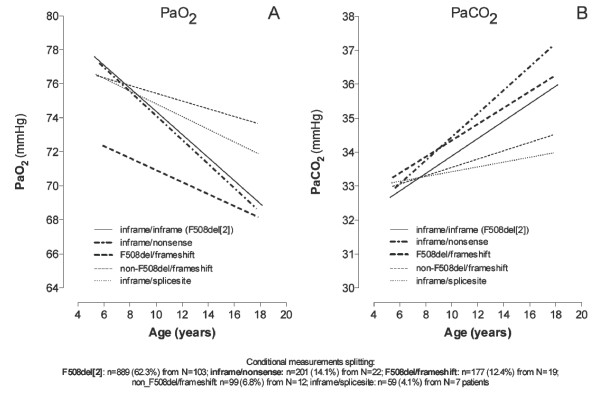
**Progression with age based on measurements of A) PaO_2 _and B) PaCO_2 _within the 5 genetic subgroups (miscellaneous excluded), obtained by linear mixed model LMM analysis**. Patient numbers are given by N, measurement numbers by n. Data distribution over age was equal for each genetic subgroup.

A potential association between gas exchange characteristics and genotypes was investigated. Data from the most frequent *CFTR *genotypes inframe/inframe (F508del[2]), inframe/nonsense mutations (F508del/R553X, F508del/G542X, F508del/Q524, F508del/E553), inframe/frameshift (mainly F508del/3905insT), non-F508del/frameshift, (mainly non-F508del/3905insT) and inframe/splicesite genotypes were incorporated as fixed effects with "age at time of annual test" as covariate, and the patient-specific intercept as a random effect. The remaining miscellaneous genotypes were excluded from the model. For all five subgroups, distribution of measurements was equally dispersed over the age range studied, and the distribution of measurements over the age range within the subgroups of genotypes is given in the additional file 1. Based on LMM analysis, Figure 2 (panel A) demonstrates that different slopes of gas exchange characteristics were found in the five genetic groups. F508del/frameshift started with the lowest PaO_2 _mean values at age 6 years (72.3 ± 1.5 mmHg), and decreased to the lowest mean values at the age of 18 years (68.1 ± 1.7 mmHg), whereas F508del[2] and inframe/nonsense showed only slightly reduced oxygenation (77.1 ± 0.67, 76.9 ± 1.4 mmHg, resp.) starting at the age of 6 years, but demonstrated the most significant deterioration over the years. Interestingly, frameshift/non_F508del and inframe/splicesite, presented with significantly milder progression (*p *< 0.05). Comparisons were significant with respect to the intercept between frame/F508del and F508del[2] (*p *< 0.0001) and inframe/nonsense (*p *< 0.005) as well as between F508del/frame and non-F508del/frame (*p *= 0.013). Otherwise, PaCO_2 _(panel B) increased from 32.9 ± 0.3 mmHg (F508del[2|), 33.1 ± 0.6 mmHg (inframe/nonsense), 33.7 ± 0.7 mmHg (frameshift), up to 35.9 ± 0.3 mmHg, 37.2 ± 0.6 mmHg, 36.0 ± 0.6, respectively, where as inframe/splicesite and non_F508del/framshift remained within the range of lower PaCO_2 _(*p *< 0.001).

### Association between Gas Exchange and Lung Function

LMM analyses was used to evaluate a potential interrelationship between gas exchange measurements over age and lung function, taking PaO_2 _as outcome measures and FRC_pleth_, LCI, V_TG_, sR_eff_, FEV_1_, FEF_50_, BMI as explanatory variables, adjusted by the "year at testing" as covariate. Table 2 shows that PaO_2 _was significantly associated with FRC_pleth _(*t*-statistics -5.575; *p *< 0.0001), FEV1 (*t*-statistics 3.451; *p *= 0.001), FEF_50 _(*t*-statistics 3.158; *p *= 0.002), and LCI (*t*-statistics -3.156; *p *= 0,002), but not with V_TG_, sR_eff_, or BMI.

**Table 2:** Univariate and multivariate linear mixed model (LMM) analysis evaluating lung function parameters as explanatory variables of PaO2, including adjustment by "year at testing"

	LMM univariate analysis adjusted for age and test year		
	**Coefficient**	**95 % CI**	**p**

FRCpleth(SDS)	-1.641	-1.907 to -1.376	< 0.0001
LCI (SDS)	-0.338	-0.403 to -0.272	< 0.0001
V_TG _(SDS)	-1.209	-1.471 to -0.947	< 0.0001
sR_eff _(SDS)	-0.517	-0.599 to -0.434	< 0.0001
FEV_1 _(SDS)	1.126	0.991 to 1.261	< 0.0001
FEF_50 _(SDS)	0.586	0.507 to 0.664	< 0.0001
BMI (SDS)	1.374	0.922 to 1.825	< 0.0001
			
	LMM multivariate analysis with backward elimination procedure*		
			
	**Coefficient**	**95 % CI**	**p**
FRCpleth(SDS)	-0.868	-1.180 to -0.5555	< 0.0001
LCI (SDS)	-0.125	-0.191 to -0.058	< 0.0001
sR_eff _(SDS)	-0.123	-0.225 to -0.021	0.019
FEV_1 _(SDS)	0.511	0.284 to 0.738	< 0.0001
FEF_50 _(SDS)	0.161	0.0412 to 0.281	0.008

* V_TG _and BMI excluded from model

**Figure 3:**
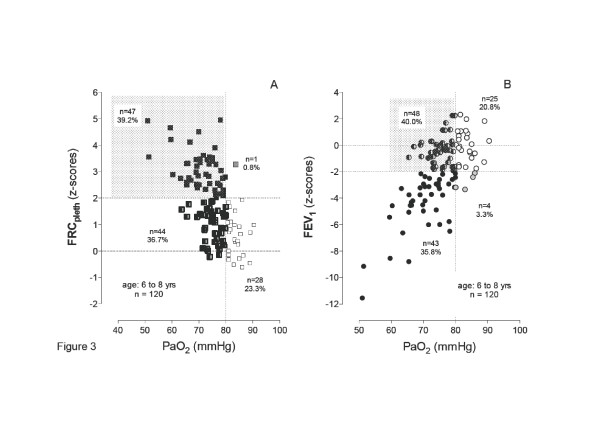
**Crosstab comparison of initial *z*-scores (mean during age range of 5 to 8 years) between A), PaO_2 _and FRC_pleth _presenting pulmonary hyperinflation in association with hypoxemia and B) PaO_2 _and FEV_1_, to present the portion of normal FEV_1 _measurements while already hypoxemic, obtained in 120 CF patients**.

**Figure 4:**
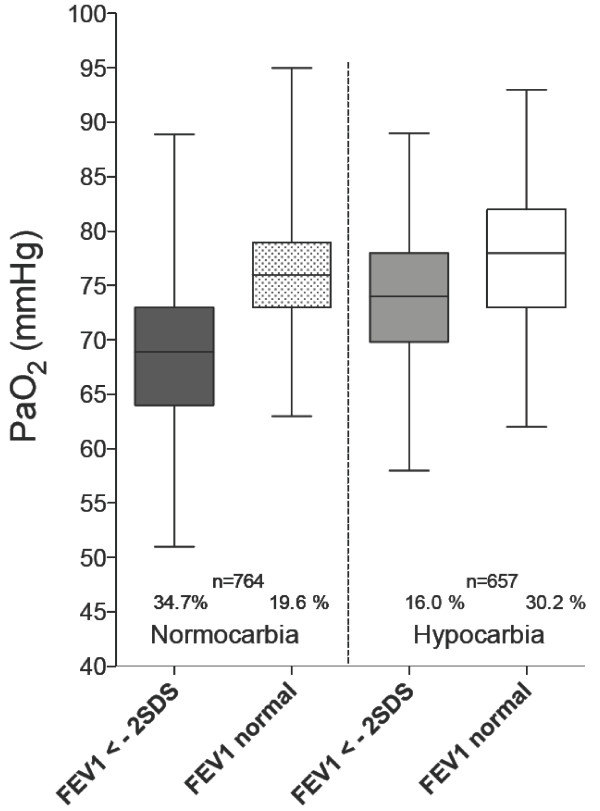
**Crosstab comparison of initial *z*-scores of FEV_1 _(mean during age range of 5 to 8 years) and oxygenation under 4 different conditions: (*i*) decreased airway patency (FEV_1 _< -2SDS) and normocarbia (PaCO_2 _> = 34 mmHg), (*ii*) normal airway patency and normocarbia, (*iii*) decreased airway patency and hypocarbia, and (*iv*) normal airway patency and hypocarbia**.

We evaluated the **early onset of abnormal lung **function in relation to oxygenation. For that purpose mean values of lung function data were computed for the age range of 5 to 8 years for each patient. 120 CF patients, who had a mean of at least 2 measurements of all lung function parameters, were eligible. Hypoxemia at rest was defined as a PaO_2 _<80 mmHg as described by Lamarre et al. [35]. Using a two-test analysis by z-score, comparisons of PaO_2 _with chi-square statistics (cross tabulation) demonstrated that the best association was found for FRC_pleth _(*Chi*-square: 21.288; *p *< 0.0001), followed by FEF_50 _(*Chi*-square: 15.579; *p *< 0.0001), sR_eff _(*Chi*-square: 11.894; *p *= 0.001), FEV_1 _(*Chi*-square: 10.33; *p *= 0.001), and LCI (*Chi*-square: 9.644; *p *= 0.002). V_TG _was not significantly associated. The association between oxygenation and pulmonary hyperinflation is presented in Figure 3 (panel A). In 39.2 % of patients hypoxemia (PaO_2 _< 80 mmHg) was associated with pulmonary hyperinflation (FRC_pleth _> 2SDS; dashed area). A further 36.7 % of patients presented with hypoxemia without pulmonary hyperinflation. As FEV_1 _is still considered to be one of the best predictors of progression in CF, we investigated whether a differentiation between normoxemia and hypoxemia can be correlated with this spirometric function parameter. Figure 3, panel B demonstrates that 40.0 % of patients presented with a normal FEV_1_, while hypoxemia was already present (dashed area). It is noteworthy that these patients already had a significant deficit in oxygenation as shown by the PaO_2_, while FEV_1 _remained within normal limits,

Under the condition of normocarbia, PaCO_2 _was mainly correlated with FEV_1 _(*p *< 0.0001), and like PaO_2, less _with V_TG _(p = 0.004) and sR_eff _(*p *= 0.003). In contrast, if hypocarbia was detected, a significant association of PaCO_2 _with FEV_1 _(*p *< 0.0001) and FEF_50 _(*p *< 0.0001) could be found, as well as with BMI (*p *= 0.011) and FRC_pleth _(*p *= 0.013). In Figure 4 stratification of measurements was performed for normocarbia *versus *hypocarbia, divided into those with normal *versus *abnormal FEV_1 _in relation to oxygenation. The question arose whether or not patients presenting with hypocarbia have an advantage with respect to oxygenation. It could be shown, that oxygenation was improved under the condition of hypocarbia, especially if forced expiratory volume was normal (*p *< 0.0001).

## Discussion

The present observational study illustrates the complexity of gas exchange in children with cystic fibrosis, especially with reference to the age-related changes in oxygenation. The age-related deterioration of oxygenation has not yet been well described, presumably due to the fact that gas exchange characteristics are not routinely evaluated over a period of a substantial number of years. Our study, performed in a representative number of cases and followed-up over a consistent number of years demonstrates that oxygenation is specifically influenced by different lung function deficits and CF genotypes. There are several major findings of this study: First, (*i*) a linear decline in oxygenation could be demonstrated over the age range 5 to 18 years, which was closely and independently related to the degree of pulmonary hyperinflation (FRC_pleth_), the degree of flow and volume limitation (FEV_1_, FEF_50_), and ventilation inhomogeneities (LCI). Secondly, (*ii*) as reported for several lung function parameters [1,2], the decline in PaO_2 _is representative of an overall deterioration of lung disease, reflecting that factors such as the degree of pulmonary hyperinflation, ventilation inhomogeneities and impeded airway function are involved in the long-term course of gas exchange characteristics. Most importantly, however, (*iii*) in more than half the patients with hypoxemia, FEV_1 _was within the range of normal values, and hence with functional deficits not detectable by spirometric lung function testing alone. Furthermore, (*iv*) an association could be found between oxygenation and the genotype. The so called "Swiss-Type" (3905insT/F508del), presented with the worst PaO_2 _values already detectable at the age of 5 years and the subgroup R553X/F508del showed the worst deterioration (steepest slope) over the age range studied. Interestingly, some special subgroups of genotypes (inframe/splicesite, 3905insT/non-F508del) showed only discrete changes in gas exchange over the years. It follows that PaO_2 _may serve as a sensitive marker of lung function deterioration in CF.

The present study demonstrates that the preservation of airway function and hence an intact static recoil of the lungs over years is essential, as has been shown by Zapletal [44]. It was reported by Hart et al. that if FEV_1 _declines in children and young adults with CF, there is an increase in the elastic load and work of breathing, resulting in a rapid shallow breathing pattern, that is associated with further impairment of gas exchange [28]. Pulmonary hyperinflation and ventilation inhomogeneities are further pathophysiologic characteristics, which have to be taken into consideration regarding progression in CF, as reported previously by our group [1,2].

### Limitations of this Study

A detailed discussion about blood gas measurements in children, the stratification into genetic subgroups, limitations in the interpretation of extensive lung function tests and the different options for statistical modeling are given in the additional file 1. An important limitation of this type of data resides in the ability to obtain repeated measurements of lung function annually over a substantial period of time. Noteworthy therefore, we were able to obtain serial annual measurements over a 10-year period in 62 % of the patients. Although the technique of blood gas measurements from the arterialized earlobe is well established and routinely used in several laboratories [26,27,32-34], in the following some technical aspects have to be mentioned. Finally, limitation of collected functional data over a wide range of years may include confounders related to changes in the technical set-up. As proposed by Soni et al [16], we assessed the influence of these potential confounders by 3 characteristics such as "year at birth", "year at diagnosis" and the "year at testing". The "year at test" proved to be the principal confounder influencing the course of PaO_2_. Most important finally, the variances of lung function over age are specific for each lung function parameter. Therefore, values of lung function data have to be expressed by z-scores, calculated from age- and gender-specific equations for value prediction for each lung function parameter, and in particular also for each lung function device as previously presented [1,2].

#### Blood gas measurements in children

Currently direct arterial catheterization of the radial artery is the widely accepted gold standard technique for obtaining the most accurate assessment of pulmonary gas exchange, especially in adult medicine. However, this technique is painful and not suitable for use in children for long-term evaluation. Alternative non-invasive methods have been proposed such as pulse oxymetry, which is used to assess arterial oxyhemoglobin saturation. However, obtained values correlated poorly with arterial PaO_2 _values [33]. Pulse oxymetry is a poor predictor of PaO_2 _because of the sigmoidal shape of the oxyhemoglobin dissociation curve and because the curve can be shifted under various clinical and physiological conditions. Therefore, capillary arterialized blood gas analysis is a very convenient technique especially suitable for children and for repeated measurements. Comparisons between earlobe capillary PaO_2 _and radial arterial PaO_2 _were performed by Godfrey et al. [26] and Gaultier et al. [27]. The former author group compared earlobe and arterial values in 8 adult subjects (age range of 26 to 63 years) at rest and on exercise, showing that the mean difference between arterial and earlobe samples for PaO_2 _at rest was 2.09 ± 2.48 mmHg and for PaCO_2 _0.65 ± 1.2. Gaultier et al. [27] studied these differences in 70 infants or children suffering from cardiac or pulmonary disease and demonstrated significant differences in PaO_2 _of 1.86 ± 0.60 mmHg only in children younger than 8 years. It was concluded that sampling blood from the earlobe is appropriate as a substitute for arterial PaO_2_, provided certain important methodological conditions such as sampling site and optimal vasodilatation are fulfilled. In a study examining PaO_2 _measurements over a long period of time, it would be preferable to validate the results periodically using arterial samples. It is, however, difficult to justify arterial blood sampling in a collective of CF children with no or only minor pulmonary function impairments. In a meta-analysis performed by Zavorsky et al. [34], it was shown that capillary blood gases accurately reflect arterial blood samples and that sampling blood from the earlobe is appropriate as an alternative to arterial PaO_2_. However, the discrepancy between capillary and arterial PaO_2 _increased with increasing PaO_2_. Reproducibility of earlobe blood gas measurements was assessed by Godfrey et al. [26] and Gaultier et al. [27].

### Oxygenation and Lung Function

There is only limited knowledge about the relationship between oxygenation and factors such as the degree of pulmonary hyperinflation, ventilation inhomogeneities and/or airway function under resting conditions in CF. Moreover, hypocarbia in CF is poorly described in the literature and the correlation of gas exchange characteristics with lung function is unknown. There is only one report in which the association between hypocarbia and hypercapnia and the matching of ventilation has been studied in dogs [45]. The present report focuses on the decline of oxygenation in CF. As demonstrated in Table 2, *oxygenation *is significantly associated with the degree of flow limitation given by the FEV_1_. This finding is in line with Hirsch et al., who could demonstrate a higher ventilatory equivalent in CF patients in comparison with control subjects, suggesting increased work of breathing due to airflow obstruction and dead space ventilation. Ventilation appears mechanically inefficient but necessary to keep arterial PaCO_2 _from rising and oxygen saturation from falling at rest [46]. Moreover, Hart et al. showed that in children and young adults with advanced stable pulmonary CF disease, falling FEV_1 _induced an increase in the respiratory muscle load, predominately by a decrease in lung compliance (C_Ldyn_) rather than an increase in total lung resistance (R_L_). Other physiologic studies have demonstrated an increase in tidal volume (V_T_) with a reduction in respiratory rate (RR) in association with impairment of gas exchange [28]. The present study indicates that oxygenation at rest is significantly associated with LCI and with FRC_pleth _and reduced forced expiratory volume (FEV_1_) as an indirect parameter of airflow limitation.

### Genotype Association with Gas Exchange Characteristics

To date, a relationship between *CFTR *genotypes and severity of pulmonary disease has proven difficult to determine [47]. The present study, however, clearly demonstrates an association between gas exchange characteristics (especially oxygenation) and genotypes. These findings are in line with previous work obtained in infants [13] and children [1,2] with CF. Schaedel et al. used FEV_1 _in terms of % predicted of normal, to demonstrate a slower rate of decline in patients with missense mutations compared with F508del homozygotes [14]. Since these patients generally had a sufficient level of pancreatic function, it was concluded that *CFTR *genotypes associated with long-term pancreatic sufficiency have more benign lung disease and better pulmonary function [12,14]. With the exception of one patient with a missense mutation, all patients in our study collective presented with pancreatic insufficiency, requiring continuous supplementation with pancreatic enzymes and high caloric nutritional support. The major new finding in the present study is an allocation of specific genotypes to (*i*) sufficient oxygenation combined with low PaCO_2 _levels, and (*ii*) insufficient oxygenation combined with normocarbia, reflecting different phenotypes of disease progression. Inframe/splicesite and non-F508del/frameshift mutations seem to have a significant better gas exchange pattern than the other groups. The different progression between F508del/frameshift and **non-**F508del/frameshift is especially striking. Based on recent findings [48] showing complete lack of *CFTR *protein at the apical membrane in F508del/3905insT compound heterozygous patients, we hypothesize that there may be an interaction between the plasma membrane resulting in a more severe phenotype. However, further experiments are needed to elucidate the fate of the 3905insT protein in the cell after its biosynthesis.

**In conclusion**, the linear decline of PaO_2 _over the years was closely associated with the degree of pulmonary hyperinflation, ventilation inhomogeneities, and parameters of airway function on the one hand and with genotypes on the other hand. We found that gas exchange characteristics (PaO_2_, PaCO_2_) are very sensitive parameters of deterioration in CF lung disease. Since the assessment of factors influencing the overall estimate of gas exchange is of major interest to understand functional deficits influencing progression not only in quantitative, but also in qualitative terms, classification into certain functional risk groups may have implications for therapeutical intervention. However, further studies are needed to demonstrate whether changes in PaCO_2 _in relation to PaO_2 _are potential predictors of exacerbation or of the long-term clinical outcome. We keep in mind, that the ability of blood gas measurements to serve as outcome measures in interventional studies largely depends from the knowledge to what extent changes recorded during a short-term study will be out of the variability of the measurement changes established by the present long term approach over years.

## Abbreviations

BMI: body mass index; BTPS: body temperature and pressure saturated; CF: Cystic Fibrosis; *CFTR*: Cystic Fibrosis Transmembrane conductance Regulator; DNA: Deoxyribonucleid Acid; FEV1: Forced Expiratory Volume in One second; FEF_50_: Forced expiratory flow at 50 percent FVC; FRC_pleth_: Functional residual capacity (plethysmographically determined); FRC_MBNW_: Functional residual capacity (determined by MBNW); FVC: Forced Vital Capacity; LCI: Lung clearance index; LMM: Linear mixed model; MBNW: Multibreath nitrogen washout; PaCO_2_: partial carbon monoxide pressure; PaO_2_: partial oxygen pressure; PA: *Pseudomonas aeruginosa*; ROC: Receiver-operated curve; SDS: Standard deviation score obtained by *z*-transformation; SEM: Standard error of the mean; sR_eff_: specific effective airway resistance; SSCP/HD: single strand confirmation polymorphism/heteroduplex; TLC: Total Lung Capacity; V_TG_: volume of trapped gas.

## Competing interests

The authors declare that they have no competing interests.

## Authors' contributions

All authors have read and approved the final manuscript. RK designed, coordinated, conceived the study and wrote all chapters; PhL took part in the interpretation of data and manuscript revision; IP participated in the data collection, and manuscript revision; PB was our consultant for statistical evaluation: SG performed the CF mutation screening, took part in the interpretation of data (especially genetics) and revising; and UF took part in the interpretation of data and revised the draft.

## Supplementary Material

Additional file 1 **Indentation of methods and data base management**. Details of methods used, robustness of the data base, limits of methods and options of statistical modelling. Click here for file

## References

[B1] Kraemer R Blum A Schibler A Ammann RA Gallati S Ventilation inhomogeneities in relation to standard lung function in patients with cystic fibrosis Am J Respir Crit Care Med 2005 171 371 378 10.1164/rccm.200407-948OC 15531750

[B2] Kraemer R Baldwin DN Ammann RA Frey U Gallati S Progression of pulmonary hyperinflation and trapped gas associated with genetic and environmental factors in children with cystic fibrosis Respir Res 2006 7 138 17137500 10.1186/1465-9921-7-138 PMC1698484

[B3] Gustafsson PM Aurora P Lindblad A Evaluation of ventilation maldistribution as an early indicator of lung disease in children with cystic fibrosis Eur Respir J 2003 22 972 979 10.1183/09031936.03.00049502 14680088

[B4] Ranganathan SC Stocks J Dezateux C Bush A Wade A Carr S Castle R Dinwiddie R Hoo AF Lum S The evolution of airway function in early childhood following clinical diagnosis of cystic fibrosis Am J Respir Crit Care Med 2004 169 928 933 10.1164/rccm.200309-1344OC 14754763

[B5] Aurora P Bush A Gustafsson P Oliver C Wallis C Price J Stroobant J Carr S Stocks J Multiple-breath washout as a marker of lung disease in preschool children with cystic fibrosis Am J Respir Crit Care Med 2005 171 249 256 10.1164/rccm.200407-895OC 15516530

[B6] Gustafsson PM Peripheral airway involvement in CF and asthma compared by inert gas washout Pediatr Pulmonol 2007 42 168 176 10.1002/ppul.20554 17186546

[B7] Beardsmore CS Lung function from infancy to school age in cystic fibrosis Arch Dis Child 1995 73 519 523 8546509 10.1136/adc.73.6.519 PMC1511453

[B8] Kraemer R Schoni MH Ventilatory inequalities, pulmonary function and blood oxygenation in advanced states of cystic fibrosis Respiration 1990 57 318 324 10.1159/000195864 2284509

[B9] Gustafsson PM Johansson HJ Dahlback GO Pneumotachographic nitrogen washout method for measurement of the volume of trapped gas in the lungs Pediatr Pulmonol 1994 17 258 268 10.1002/ppul.1950170410 8208598

[B10] Lum S Gustafsson P Ljungberg H Hulskamp G Bush A Carr SB Castle R Hoo AF Price J Ranganathan S Early detection of cystic fibrosis lung disease: multiple-breath washout versus raised volume tests Thorax 2007 62 341 347 10.1136/thx.2006.068262 17121870 PMC2092467

[B11] Ranganathan SC Dezateux C Bush A Carr SB Castle RA Madge S Price J Stroobant J Wade A Wallis C Stocks J Airway function in infants newly diagnosed with cystic fibrosis Lancet 2001 358 1964 1965 10.1016/S0140-6736(01)06970-7 11747924

[B12] Corey M Edwards L Levison H Knowles M Longitudinal analysis of pulmonary function decline in patients with cystic fibrosis J Pediatr 1997 131 809 814 10.1016/S0022-3476(97)70025-8 9427882

[B13] Kraemer R Birrer P Liechti-Gallati S Genotype-phenotype association in infants with cystic fibrosis at the time of diagnosis Pediatr Res 1998 44 920 926 10.1203/00006450-199812000-00016 9853928

[B14] Schaedel C de Monestrol I Hjelte L Johannesson M Kornfalt R Lindblad A Strandvik B Wahlgren L Holmberg L Predictors of deterioration of lung function in cystic fibrosis Pediatr Pulmonol 2002 33 483 491 10.1002/ppul.10100 12001283

[B15] Wagener JS Taussig JM Burrows B Hernried L Boat T Sturgess JM Comparison of lung function and survival patterns between cystic fibrosis and emphysema of chronic bronchitis patients Perspectives in Cystic Fibrosis 1980 Mississanga, Ontario: Imperial Press 236-245-236-240

[B16] Soni R Dobbin CJ Milross MA Young IH Bye PP Gas exchange in stable patients with moderate-to-severe lung disease from cystic fibrosis J Cyst Fibros 2008 7 285 291 10.1016/j.jcf.2007.11.003 18785322

[B17] Warwick WJ Pogue RE Gerber HU Nesbitt CJ Survival patterns in cystic fibrosis J Chronic Dis 1975 28 609 622 10.1016/0021-9681(75)90074-0 1239460

[B18] Rosenstein BJ Cutting GR The diagnosis of cystic fibrosis: a consensus statement. Cystic Fibrosis Foundation Consensus Panel J Pediatr 1998 132 589 595 10.1016/S0022-3476(98)70344-0 9580754

[B19] Liechti-Gallati S Schneider V Neeser D Kraemer R Two buffer PAGE system-based SSCP/HD analysis: a general protocol for rapid and sensitive mutation screening in cystic fibrosis and any other human genetic disease Eur J Hum Genet 1999 7 590 598 10.1038/sj.ejhg.5200338 10439967

[B20] Bennett LC Kraemer R Liechti-Gallati S Buccal cell DNA analysis in premature and term neonates: screening for mutations of the complete coding region for the cystic fibrosis transmembrane conductance regulator Eur J Pediatr 2000 159 99 102 10.1007/PL00013814 10653340

[B21] Kraemer R Delosea N Ballinari P Gallati S Crameri R Effect of allergic bronchopulmonary aspergillosis on lung function in children with cystic fibrosis Am J Respir Crit Care Med 2006 174 1211 1220 10.1164/rccm.200603-423OC 16959918

[B22] Kraemer R Meister B Fast real-time moment-ratio analysis of multibreath nitrogen washout in children Journal of applied physiology: respiratory, environmental and exercise physiology 1985 59 1137 1144 10.1152/jappl.1985.59.4.1137 4055593

[B23] Kraemer R Zehnder M Meister B Intrapulmonary gas distribution in healthy children Respir Physiol 1986 65 127 137 10.1016/0034-5687(86)90045-9 3764118

[B24] Zapletal A Samanek M Paul T Lung function in children and adolescents 1987 Basel (Switzerland): Karger

[B25] Manzke H Stadlober E Schellauf HP Combined body plethysmographic, spirometric and flow volume reference values for male and female children aged 6 to 16 years obtained from "hospital normals" Eur J Pediatr 2001 160 300 306 10.1007/s004310100724 11388599

[B26] Godfrey S Wozniak ER Courtenay Evans RJ Samuels CS Ear lobe blood samples for blood gas analysis at rest and during exercise Br J Dis Chest 1971 65 58 64 5110177

[B27] Gaultier C Boule M Allaire Y Clement A Buvry A Girard F Determination of capillary oxygen tension in infants and children: assessment of methodology and normal values during growth Bull Eur Physiopathol Respir 1979 14 287 297 476326

[B28] Hart N Polkey MI Clement A Boule M Moxham J Lofaso F Fauroux B Changes in pulmonary mechanics with increasing disease severity in children and young adults with cystic fibrosis Am J Respir Crit Care Med 2002 166 61 66 10.1164/rccm.2112059 12091172

[B29] Hart N Tounian P Clement A Boule M Polkey MI Lofaso F Fauroux B Nutritional status is an important predictor of diaphragm strength in young patients with cystic fibrosis Am J Clin Nutr 2004 80 1201 1206 15531666 10.1093/ajcn/80.5.1201

[B30] Fauroux B Nicot F Essouri S Hart N Clement A Polkey MI Lofaso F Setting of noninvasive pressure support in young patients with cystic fibrosis Eur Respir J 2004 24 624 630 10.1183/09031936.04.0000137603 15459142

[B31] Fauroux B Webb AK, Ratjen FA Nonivasive ventilation in cystic fibrosis Cystic Fibrosis 2006 35 Wakefield, UK: European Respiratory Society; Eur Respir Mon 127 138

[B32] MacIntyre J Norman JN Smith G Use of capillary blood in measurement of arterial PO2 Br Med J 1968 3 640 643 5673210 10.1136/bmj.3.5619.640 PMC1986564

[B33] Pitkin AD Roberts CM Wedzicha JA Arterialised earlobe blood gas analysis: an underused technique Thorax 1994 49 364 366 8202909 10.1136/thx.49.4.364 PMC475372

[B34] Zavorsky GS Cao J Mayo NE Gabbay R Murias JM Arterial versus capillary blood gases: a meta-analysis Respir Physiol Neurobiol 2007 155 268 279 10.1016/j.resp.2006.07.002 16919507

[B35] Lamarre A Reilly BJ Bryan AC Levison H Early detection of pulmonary function abnormalities in cystic fibrosis Pediatrics 1972 50 291 298 5045356

[B36] Stokes DC Wohl ME Khaw KT Strieder DJ Postural hypoxemia in cystic fibrosis Chest 1985 87 785 789 10.1378/chest.87.6.785 3996068

[B37] Wolf B Gaultier C Lopez C Boule M Girard F Hypoxemia in attack free asthmatic children: relationship with lung volumes and lung mechanics Bull Eur Physiopathol Respir 1983 19 471 476 6640166

[B38] Prader A Largo R Molinari L Issler C Physical growth of Swiss children from birth to 20 years of age Helv Paediatr Acta 1989 52 1 125 2737921

[B39] Cole TJ Freeman JV Preece MA British 1990 growth reference centiles for weight, height, body mass index and head circumference fitted by maximum penalized likelihood Stat Med 1998 17 407 429 10.1002/(SICI)1097-0258(19980228)17:4<407::AID-SIM742>3.0.CO;2-L 9496720

[B40] Steiner B Truninger K Sanz J Schaller A Gallati S The role of common single-nucleotide polymorphisms on exon 9 and exon 12 skipping in nonmutated CFTR alleles Hum Mutat 2004 24 120 129 10.1002/humu.20064 15241793

[B41] Laird NM Donnelly C Ware JH Longitudinal studies with continuous responses Stat Methods Med Res 1992 1 225 247 10.1177/096228029200100302 1341659

[B42] Brown H Prescott R Applied Mixed Models in Medicine 2006 2 Chichester, West Sussex, Enland: John Wiley & Sons Ltd

[B43] Norusis MJ SPSS Statistics 17.0 Advanced Statistical Procedures Companion 2008 Prentice Hall Inc.; New York

[B44] Zapletal A Desmond KJ Demizio D Coates AL Lung recoil and the determination of airflow limitation in cystic fibrosis and asthma Pediatr Pulmonol 1993 15 13 18 10.1002/ppul.1950150103 8419893

[B45] Domino KB Swenson ER Hlastala MP Hypocapnia-induced ventilation/perfusion mismatch: a direct CO2 or pH-mediated effect? Am J Respir Crit Care Med 1995 152 1534 1539 7582289 10.1164/ajrccm.152.5.7582289

[B46] Hirsch JA Zhang SP Rudnick MP Cerny FJ Cropp GJ Resting oxygen consumption and ventilation in cystic fibrosis Pediatr Pulmonol 1989 6 19 26 10.1002/ppul.1950060107 2704578

[B47] Kerem E Corey M Kerem BS Rommens J Markiewicz D Levison H Tsui LC Durie P The relation between genotype and phenotype in cystic fibrosis-analysis of the most common mutation (delta F508) N Engl J Med 1990 323 1517 1522 2233932 10.1056/NEJM199011293232203

[B48] Sanz J von Kanel T Schneider M Steiner B Schaller A Gallati S The CFTR frameshift mutation 3905insT and its effect at transcript and protein level Eur J Hum Genet 2009 19724303 10.1038/ejhg.2009.140 PMC2987192

