# Supplementary material for: Long-term gas exchange characteristics as markers of deterioration in patients with cystic fibrosis
Source: Respir Res. 2009 Nov 12;10(1):106. doi: 10.1186/1465-9921-10-106 (PMC2780404; doi:10.1186/1465-9921-10-106)
Supplement: Additional file 1 — Indentation of methods and data base management. Details of methods used, robustness of the data base, limits of methods and options of statistical modelling. [file 1465-9921-10-106-S1.doc]

Additional file 1

Indentation of methods and data base management
related to the main text of: Long-term gas exchange characteristics as markers of deterioration in patients with cystic fibrosis
*Richard Kraemer, Philipp Latzin, Isabelle Pramana, Pietro Ballinari, Sabina Gallati, and Urs Frey*

**Methods**

***Pulmonary Function Measurements***

Whole-body plethysmography and the multibreath nitrogen washout (MBNW) technique provided data pertaining to functional residual capacity (FRC_pleth_, FRC_MBNW_), lung clearance index (LCI), volume of trapped gas (V_TG_), effective specific airway resistance (sR_eff_), and forced expiratory indices (FEV_1_, FEF_50_). Airway resistance was also recorded during quiet breathing (no panting). Measurement techniques have been described in detail in previous papers [1-3]. All values were expressed as a standard deviation score (SDS) based on gender- and age-specific regression equations [4-7], specifically calculated for each lung function device as previously presented [1, 3].

Whole body plethysmography consisted in using a volume-constant, pressure-variable plethysmograph with an air bag body temperature and pressure saturated (BTPS) compensation unit (BodyScreen, Jaeger Würzburg, Germany) until December 4, 1993. Thereafter, the MasterLab plethysmograph was employed (MasterLab, Jaeger Würzburg, Germany). This instrument uses electronic BTPS-compensation.

The sequences of test measurements were as follows: After blood gas measurements under ambient air, and prior to plethysmographic measurements, resting end-expiratory lung volume (FRC_MBNW_) and quantification of ventilation inhomogeneities (LCI) were determined by open-circuit multibreath nitrogen washout (MBNW) technique [4] using the Paediatric Pulmonary Unit (SensorMedics 2200, Yorba Linda, Ca, USA). Technical details for this procedure have been reported previously [1]. Zero point adjustment and two-point calibration were performed and patients initiated tidal breathing in ambient air whilst seated and wearing a nose clip. Tidal breathing was monitored until a constant resting end-expiratory level was obtained. The inspiratory port was then automatically switched to 100 % O_2_ and the washout was continued until an end-tidal fraction of nitrogen (F_N2_) was 0.02. The breathing pattern, registered during the resting phase before starting the multibreath washout, was quantified in terms of respiratory rate, tidal volume (V_T_), minute ventilation (V_E_), and the V_T_/FRC_MBNW_ ratio.

Thereafter the children were closed into the plethysmograph and requested to breathe at a coached normal frequency during shutter closure for measurements of FRC_pleth_. Airway resistances were also recorded during quiet breathing (no panting) under strict BTPS conditions. The current study quantifies resistance obtained from the regression slope using specific effective airway resistance (sR_eff_) [7]. This value represents the average volume-independent resistance calculated using concomitant changes in the volume-pressure and flow-pressure relationship obtained throughout the entire respiratory cycle. Calculation of sR_eff_ is based on the definition of resistive work of breathing. The total work of breathing is the integral of the pressure in the lung (p_L_) over volume for the whole breathing cycle:

where p_L_ is determined by the volume-change of the box signal and the volume of the lung (FRC_pleth_).

∆V is the volume change in the body box, P_amb_ the ambient pressure and FRC_pleth_ the end-expiratory volume. The volume changes are measured relative to an arbitrary start volume, start pressure (P_0_), where p_L_ is the sum of the p_0_, the elastic recoil component (E), defined as ∆p_L_ / ∆V and the resistive pressure R.V’. It follows:

when integrated over a whole breathing cycle and assuming that lung volume remains constant between beginning and end of the breathing cycle. Moreover,

when integrated over a whole breathing cycle and assuming that lung volume remains constant between beginning and end of the breathing cycle.

Therefore, the resistive component can be ignored and we obtain

This yields the definition of the specific effective resistance:

The integral $\oint D\text{VdV}$ is equivalent to the area enclosed by the specific work of breathing loop and the integral $\oint V'\text{dV}$ is equivalent to the area of the flow / volume-loop. Both loops are measured in real-time during tidal breathing and the ratio of these two integrals is equivalent to calculating the slope through all (not just two) flow-volume loop sample points.

Following a short rest period, indexes of forced expiratory air flow limitation including forced expired volume in one second (FEV_1_) and maximal expired volume at 50 percent of forced vital capacity (FEF_50_) were calculated from maximal expiratory flow volume curves. All measurements were stored for offline analysis and the 3-5 technically most satisfactory manoeuvres were chosen for analysis using a computer system adapted for children.

Data were analyzed in order to examine (*a*) annual changes in gas exchange characteristics (PaO_2_, PaCO_2_) with age, (*b*) the corresponding functional residual capacity measured by whole-body plethysmography (FRC_pleth_) as a measure of pulmonary hyperinflation, (*c*) the lung clearance index as a measure of ventilation inhomogeneities, (*d*) the volume of trapped gases (V_TG_=FRC_pleth_-FRC_MBNW_) [8-10], and (*e*) characteristics of gas exchange in relation to the degree of bronchial obstruction. This was evaluated using effective specific airway resistance (sR_eff_) [7, 11] as a measure of airway narrowing during quiet breathing, and forced expiratory volume in one second (FEV_1_) and maximal expiratory flow at 50% of vital capacity (FEF_50_) as a measure of airflow limitation.

***Blood Gas Analysis***

In children, the preferred technique for routine blood gas analysis is the sampling of arterialized capillary blood from the earlobe [10, 12, 13], a technique that has been established for clinical use, and applied in various long-term studies [14-17]. The accuracy of such technique depends upon careful preparation of the earlobe,[12] puncture technique [12, 13] and immediate analysis. Oxygen (PaO_2_) and carbon dioxide (PaCO_2_) tensions were measured in arterialized blood collected from the ear lobe [10, 12, 13, 18] using a Radiometer ABL5, Copenhagen, Denmark. Several author groups have proven accuracy of PaO_2_, PaCO_2_ and pH measured in arterialized capillary blood taken from the earlobe for clinical and long-term evaluation of gas exchange [12, 13, 18-20], provided certain important methodological conditions are fulfilled.

Great care was taken to prepare the ear lobe blood of the children in a quiet atmosphere according to the technique initially described [12, 13] and previously established in our laboratory [10]; most of patients being familiar with the well-known test procedure. Prior to the blood draw, vasodilatation of the earlobe was achieved by rubbing the lobe with a nicotinate paste (Finalgon) [12, 13, 18, 19] for at least 10 minutes and heating with an infrared lamp. The arterialized blood was collected from a drop on the inferior aspect of the earlobe, from which it was drawn into 3 thin heparinized glass capillary tubes by surface tension under the guidance of a gloved finger over the open end of the tube. The capillary tubes were then kept on ice until aspiration into the gas analyzer, which was carried out immediately after the blood draw.

***Definition of Hypocarbia***

Low level of PaCO_2_ suggesting that a considerable number of patients pass especially in younger ages a phase of hypocarbia, three different statistical approaches were chosen to define a potential threshold for a hypocarbic condition. Apart from a ROC and linear discriminant analysis, we used binary logistic regression dichotomizing the measurements into 2 subgroups. Taking FEV_1_ and FRC_pleth_ as lung function parameters with the highest association with the gas exchange characteristics, one subgroup was defined as the blood gas measurements with normal lung function (FEV_1_ > -2 SDS and FRC_pleth_ < 2 SDS), and the other subgroup consisted of blood gas measurements und abnormal lung function (FEV_1_ < -2 SDS and FRC_pleth_ > 2 SDS). Using this procedure, the lower level of PaCO_2_ to be measured under the condition of normal lung function was defined according the equation (PaCO_2_ = 0.339*Ind-11.730), where Ind was the index of normal (=0) or abnormal (=1) lung function. It follows that z = -11.730+(0.339*PaCO_2_) and accordingly, the threshold can be calculated according to PaCO_2_=11.730/0.339 = 34.6 mmHg. With an accuracy of 68.2% and an area under the curve of 0.707, the threshold of a PaCO_2_ of 34 mmHg was confirmed by the ROC analysis. The linear discriminant analysis revealed a threshold value of 33.8 mmHg, calculated from the canonical discriminant function coefficients and 33.9 mmHg calculated from Fisher’s linear discriminant function coefficients respectively. For the present study, therefore, hypocarbia was defined as a PaCO_2_ less or equal to 34 mmHg.

***Data Computation and Statistical Evaluation***

Repeated measurements of lung function data were calculated as mean±SEM annual changes for visual presentation, and the statistical analysis included procedures such as ANOVA, linear mixed model (LMM) analysis,[21] and binary logistic regression analysis, using SPSS (version 17, SPSS Inc., Chicago, USA). Graphical presentation was completed using Prism software (version 4.0, GraphPad Software, Inc., San Diego, USA).

Linear mixed models (LMM) analysis was used to assess the relationship between gas exchange characteristics (PaO_2_, PaCO_2_) as dependent variables, group characteristics (confounders, predisposing factors, genotype) as fixed factors, and each lung function parameter (FRC_pleth_, LCI, V_TG_, sR_eff_, FEV_1_, FEF_50_) as well as age at test as covariates. This technique is suitable for analyzing the association between time and covariates from irregularly spaced serial data from individuals (i.e. repeated measurements), without being affected by missing data.[1-3, 21, 22] All lung function parameters and breathing characteristics were expressed as standard deviation scores (SDS), if not given as ratio calculated from original absolute data. This procedure permitted modelling of mean values, variances and co-variances. The analysis covered an age range of 6 to 18 years. Differences between lung function parameters within preformed gas exchange groups over time were also assessed by *t*-test, evaluating differences of the regression slopes between groups, setting in turn each group to zero. Results with p<0.05 were considered statistically significant. The interrelationship between gas exchange measurements and lung function was evaluated by a multivariable regression model with backward procedure, taking PaO_2_ and PaCO_2_ values as outcome measures, and FRC_pleth_, LCI, V_TG_, sR_eff_, FEV_1_, FEF_50_ as explanatory variables. Binary logistic regression procedure was used in order to predict the presence or absence of characteristics such as normal gas exchange, hypoxemia, and hypocarbia taken as a dichotomous dependent variable.

**Results**

***Robustness of Database and Search for Potential Confounders***

In order to take into consideration potential confounding factors such as age at

diagnosis, improvement of management and/or treatment over the years of observation as described by Soni et al. [23], we assessed the influence of 3 confounders: “birth year”, “year at diagnosis” and the “year at testing” aimed to depict a potential “secular trend” [1], as it has been described in other fields of medical research [24, 25]. For that purpose we produced partial correlation coefficients describing the relationship between PaO_2_ and the confounder variables, while adjusting for the effects of the “age at lung function testing”. The Pearson product-moment correlation matrix revealed that all the 3 confounders variables are highly inter-correlated (*p*<0.0001), all 3 were also significantly correlated with PaO_2_ (*p*<0.0001). Using LMM-analysis and adding FRC_pleth_ as the lung function parameter most closely correlated to PaO_2_ (**Table 2**) into the model, we revealed that “year at testing” was the most significant confounder of oxygenation (*t*=2.831, *p*=0.005) and, therefore, most suitable to represent the potential secular trend. Consequently, and in order to guarantee the robustness of the database, “year at testing” was taken as a covariant in all statistical analyses.

***Gas exchange follow-up data in CF patients who died***

Some clinicians could be interested to know whether the initial gas exchange characteristics or subsequent deterioration over time could predict poorer outcomes. The individual follow-up data of these CF patients who died during the observation period are given in the **Figure**. It can be shown that primarily oxygenation is hampered and hypercapnia was only present in some end-stage conditions. However, comparing individual gas exchange data with the corresponding age related to marginal means of the surviving collective demonstrated significant differences for PaO_2_ (*p*=0.001) and PaCO_2_ (*p*=0.007), indicating that gas exchange characteristics measured over time, could well serve as outcome parameters.

**Discussion**
***Limitations of this Study***

Certain statistical issues related to topics such as stratification into subgroups, repeated measurements, definitions and threshold criteria as well as confounders always leave room for discussion since patients with a disease such as CF often have a very heterogeneous presentation. The current study represents a very large patient cohort, many of whom were followed up over a long period of time. Although we were able to obtain serial annual measurements over a 10-year period in 62 % of the patients, an important limitation of this type of data, however, resides in the ability to obtain repeated measurements of lung function annually over a substantial period of time. Linear mixed-effects model analysis provides an ideal statistical method for the interpretation of repeated measurement data such as these, particularly when repeated testing results in small proportions of incomplete data.

**Blood gas measurements in children**. The technical details in relation to capillary blood sampling, which have strictly to be respected, are given in the method section, and the limitations of this technique is given in the main document.

**Control group and predicting equations.** One weakness in this study could be seen in the lack of locally collected control data from healthy children. However, the built-up of such a control group within an observational study is hardly feasible. Therefore, *z*-transformation has to be performed on the basis of prediction equations. For the multibreath nitrogen washout data computation was performed on own prediction formulas [5], and spirometric and whole-body plethysmographic data were analyzed according the equation given as appendix file to previous publication [1] to be found under <http://ajrccm.atsjournals.org/cgi/data/171/4/371/DC1/1>

**Stratification of specific *CFTR* genotypes** required a frequency-based approach to search for significant associations. Although the numbers of patients within the second-frequent and third-frequent subgroups are by nature small, they were all equally distributed over the age-range investigated. The distribution of measurements over the age range within the subgroups of genotypes of genotypes is given in the following Table.
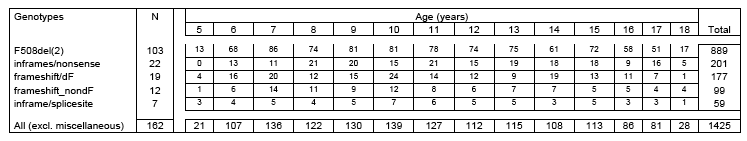


The complexity of the PaO_2_ and PaCO_2_ course related to age in CF patients, and its relationship to predisposing and confounding factors necessitated evaluation of these data using advanced statistical methods. Fixed effect covariances and randomized block designs of data with non-constant variability and missing data were best analyzed by linear mixed-effect (LMM) procedures, also known as hierarchical linear models. The adequacy of stratification within genetic subgroups is critical to the interpretation of differences observed in outcome measures between each subgroup. Possible categorizations might include F508del versus non-F508del, or in terms of molecular mechanisms and consequences observed at the gene or protein level. Our cohort study represents primarily the *CFTR* genotype distribution for Switzerland, where a substantial proportion of patients carry the severe CF mutation (3905insT/F508del) associated with the highest mortality rate [10, 26-28]. While stratification of gas exchange subgroups was adequate, stratification of specific *CFTR* genotypes required a frequency-based approach to search for significant associations.

**Influence of treatment**. Another potential confounder was the factor of individual differences in treatment, which could not be included within the scope of this report. Nevertheless, it is worth noting that the Bernese Centre closely followed the international guidelines of the North American CF Association, the European Working Group for CF and hence the European CF Society [29, 30]. Therefore, the present study can only report on disease progression in a collective of CF patients studied over 13 years and in whom both medical and paramedical therapeutic regimens continued to improve as a whole. Moreover, the present study is based on a prospectively conducted database, through which analysis was performed in order to determine the discriminative power of different lung function parameters and gas exchange characteristics collected serially.

**Options of Statistical Modelling.** The complexity of the PaO_2_ and PaCO_2_ course with age in CF patients, and its relationship to predisposing and confounding factors necessitated evaluation of these data using advanced statistical methods. Fixed effect covariances and randomized block designs of data with non-constant variability and missing data were best analyzed by Linear Mixed-effect Model (LMM) procedures, also known as hierarchical linear models. The choice to use LMM analysis in preference to General Linear Models (GLM) was based on the consideration that the data to be statistically analyzed in the present study include (*i*) fixed and random effects, (*ii*) repeated measures at different time points, and (*iii*) missing data. Mixed models analysis of repeated measures has several advantages. The time points at which measurements are obtained need not be constant for all subjects. Subjects with incomplete observations can be included in the analysis if at least a certain number of measurements are available, in our case at least 3 in each patient. There are a few data analysis situations where LMM and GLM analysis will provide identical results (e.g., fixed effects ANOVA with only one random component, the residual variance) and these designs are good starting points to demonstrate their similarities. However, since LMM can handle multiple sources of variation better than GLM, mixed models have more flexibility to make broader and more appropriate inferences about data. If there is a randomized block design or any design that includes two or more random effects - including repeated measures designs - then versatility of LMM should be considered. The default procedure with LMM estimates variance components through Restricted-Maximum Likelihood (REML) while GLM employs moment estimates. For this reason, LMM is much more computationally intensive than GLM, which implies certain models and may take a longer time to fit.

**References**

1. Kraemer R, Blum A, Schibler A, Ammann RA, Gallati S: **Ventilation inhomogeneities in relation to standard lung function in patients with cystic fibrosis.** *Am J Respir Crit Care Med* 2005, **171:**371-378.

2. Kraemer R, Delosea N, Ballinari P, Gallati S, Crameri R: **Effect of allergic bronchopulmonary aspergillosis on lung function in children with cystic fibrosis.** *Am J Respir Crit Care Med* 2006, **174:**1211-1220.

3. Kraemer R, Baldwin DN, Ammann RA, Frey U, Gallati S: **Progression of pulmonary hyperinflation and trapped gas associated with genetic and environmental factors in children with cystic fibrosis.** *Respir Res* 2006, **7:**138.

4. Kraemer R, Meister B: **Fast real-time moment-ratio analysis of multibreath nitrogen washout in children.** *Journal of applied physiology: respiratory, environmental and exercise physiology* 1985, **59:**1137-1144.

5. Kraemer R, Zehnder M, Meister B: **Intrapulmonary gas distribution in healthy children.** *Respir Physiol* 1986, **65:**127-137.

6. Zapletal A, Samanek M, Paul T: *Lung function in children and adolescents.* Basel (Switzerland): Karger; 1987.

7. Manzke H, Stadlober E, Schellauf HP: **Combined body plethysmographic, spirometric and flow volume reference values for male and female children aged 6 to 16 years obtained from "hospital normals".** *Eur J Pediatr* 2001, **160:**300-306.

8. Matthys H, Keller R, Herzog H: **Plethysmographic assessment of trapped air in man.** *Respiration* 1970, **27:**447-461.

9. Logvinoff M, Mossay C, Geubelle F: **Oxygen tension in arterialized cutaneous blood and lung function in asthmatic children.** *Acta Paediatr Belg* 1976, **29:**239-243.

10. Kraemer R, Schoni MH: **Ventilatory inequalities, pulmonary function and blood oxygenation in advanced states of cystic fibrosis.** *Respiration* 1990, **57:**318-324.

11. Kraemer R, Blum A, Schibler A, Ammann RA, Gallati S: **Ventilation Inhomogeneities in Relation to Standard Lung Function in Patients with Cystic Fibrosis.** *Am J Respir Crit Care Med* 2004 Nov 5; [Epub ahead of print].

12. Godfrey S, Wozniak ER, Courtenay Evans RJ, Samuels CS: **Ear lobe blood samples for blood gas analysis at rest and during exercise.** *Br J Dis Chest* 1971, **65:**58-64.

13. Gaultier C, Boule M, Allaire Y, Clement A, Buvry A, Girard F: **Determination of capillary oxygen tension in infants and children: assessment of methodology and normal values during growth.** *Bull Eur Physiopathol Respir* 1979, **14:**287-297.

14. Hart N, Polkey MI, Clement A, Boule M, Moxham J, Lofaso F, Fauroux B: **Changes in pulmonary mechanics with increasing disease severity in children and young adults with cystic fibrosis.** *Am J Respir Crit Care Med* 2002, **166:**61-66.

15. Hart N, Tounian P, Clement A, Boule M, Polkey MI, Lofaso F, Fauroux B: **Nutritional status is an important predictor of diaphragm strength in young patients with cystic fibrosis.** *Am J Clin Nutr* 2004, **80:**1201-1206.

16. Fauroux B, Nicot F, Essouri S, Hart N, Clement A, Polkey MI, Lofaso F: **Setting of noninvasive pressure support in young patients with cystic fibrosis.** *Eur Respir J* 2004, **24:**624-630.

17. Fauroux B: **Nonivasive ventilation in cystic fibrosis.** In *Cystic Fibrosis.* Edited by Webb AK, Ratjen FA. Wakefield, UK: European Respiratory Society; 2006: Eur Respir Mon. 35,127-138

18. Pitkin AD, Roberts CM, Wedzicha JA: **Arterialised earlobe blood gas analysis: an underused technique.** *Thorax* 1994, **49:**364-366.

19. MacIntyre J, Norman JN, Smith G: **Use of capillary blood in measurement of arterial PO2.** *Br Med J* 1968, **3:**640-643.

20. Zavorsky GS, Cao J, Mayo NE, Gabbay R, Murias JM: **Arterial versus capillary blood gases: a meta-analysis.** *Respir Physiol Neurobiol* 2007, **155:**268-279.

21. Laird NM, Donnelly C, Ware JH: **Longitudinal studies with continuous responses.** *Stat Methods Med Res* 1992, **1:**225-247.

22. Corey M, Edwards L, Levison H, Knowles M: **Longitudinal analysis of pulmonary function decline in patients with cystic fibrosis.** *J Pediatr* 1997, **131:**809-814.

23. Soni R, Dobbin CJ, Milross MA, Young IH, Bye PP: **Gas exchange in stable patients with moderate-to-severe lung disease from cystic fibrosis.** *J Cyst Fibros* 2008, **7:**285-291.

24. Laursen EM, Koch C, Petersen JH, Muller J: **Secular changes in anthropometric data in cystic fibrosis patients.** *Acta Paediatr* 1999, **88:**169-174.

25. Girou E, Brun-Buisson C, Taille S, Lemaire F, Brochard L: **Secular trends in nosocomial infections and mortality associated with noninvasive ventilation in patients with exacerbation of COPD and pulmonary edema.** *Jama* 2003, **290:**2985-2991.

26. Liechti-Gallati S, Bonsall I, Malik N, Schneider V, Kraemer LG, Ruedeberg A, Moser H, Kraemer R: **Genotype/phenotype association in cystic fibrosis: analyses of the delta F508, R553X, and 3905insT mutations.** *Pediatr Res* 1992, **32:**175-178.

27. Kraemer R, Birrer P, Liechti-Gallati S: **Genotype-phenotype association in infants with cystic fibrosis at the time of diagnosis.** *Pediatr Res* 1998, **44:**920-926.

28. Gallati S: **Genetics of cystic fibrosis.** In *Cystic fibrosis and bronchiectasis.* *Volume* 24. Edited by Lynch JP. New York, NY: Thieme Medical Publishers, Inc.; 2003: 629-637.[Rubin BK (Series Editor): *Seminars in Respiratory and Critical Care Medicine*].

29. Doring G, Conway SP, Heijerman HG, Hodson ME, Hoiby N, Smyth A, Touw DJ: **Antibiotic therapy against Pseudomonas aeruginosa in cystic fibrosis: a European consensus.** *Eur Respir J* 2000, **16:**749-767.

30. Gibson RL, Burns JL, Ramsey BW: **Pathophysiology and management of pulmonary infections in cystic fibrosis.** *Am J Respir Crit Care Med* 2003, **168:**918-951.
